# Bias through selective inclusion and attrition: Representativeness when comparing provider performance with routine outcome monitoring data

**DOI:** 10.1002/cpp.2364

**Published:** 2019-04-23

**Authors:** Edwin de Beurs, Lisanne Warmerdam, Jos Twisk

**Affiliations:** ^1^ Clinical Psychology Leiden University Leiden The Netherlands; ^2^ Research Department Stichting Benchmark GGZ Bilthoven The Netherlands; ^3^ Methodology and Applied Biostatistics Vrije Universiteit Amsterdam Amsterdam The Netherlands

**Keywords:** attrition bias, imputation, propensity weighting, response rate, selection bias

## Abstract

**Background:**

Observational research based on routine outcome monitoring is prone to missing data, and outcomes can be biased due to selective inclusion at baseline or selective attrition at posttest. As patients with complete data may not be representative of all patients of a provider, missing data may bias results, especially when missingness is not random but systematic.

**Methods:**

The present study establishes clinical and demographic patient variables relevant for representativeness of the outcome information. It applies strategies to estimate sample selection bias (weighting by inclusion propensity) and selective attrition bias (multiple imputation based on multilevel regression analysis) and estimates the extent of their impact on an index of provider performance. The association between estimated bias and response rate is also investigated.

**Results:**

Provider‐based analyses showed that in current practice, the effect of selective inclusion was minimal, but attrition had a more substantial effect, biasing results in both directions: overstating and understating performance. For 22% of the providers, attrition bias was estimated to be in excess of 0.05 ES. Bias was associated with overall response rate (*r* = .50). When selective inclusion and attrition bring providers' response below 50%, it is more likely that selection bias increased beyond a critical level, and conclusions on the comparative performance of such providers may be misleading.

**Conclusions:**

Estimates of provider performance were biased by selection, especially by missing data at posttest. Results on the extent and direction of bias and minimal requirements for response rates to arrive at unbiased performance indicators are discussed.

**Key Practitioner Message**

Unbiased performance indicators require sufficient ROM response rates.

## 1 INTRODUCTION

In the Netherlands, routine outcome monitoring (ROM) is implemented to support individual treatments in mental health services (MHS) by informing therapists and patients on the progress made (de Beurs et al., 2011; Lambert, 2007). In addition, aggregated data from ROM are used to evaluate and improve the quality of MHS (de Beurs, Barendregt, & Warmerdam, 2017), in line with international efforts to do the same (Kilbourne et al., 2018; Porter, Kaplan, & Frigo, 2017).

In 2006, performance appraisal became important when the Dutch government introduced a new health insurance act for regulated competition among providers and among health insurers (Enthoven & van de Ven, 2007). Quality assessment is of key importance, as providers are supposed to compete on quality and efficiency, insurers should purchase care based on price and performance, and patients are expected to make an informed choice for those providers with the best outcomes. The new legislation aimed to counteract ever‐rising health care costs and simultaneously improve quality by increasing transparency about costs and outcomes.

The use of outcome data to monitor, evaluate, and learn from the performance of mental health care providers is called benchmarking (Bayney, 2005). A benchmarking institute, Stichting Benchmark Geestelijke GezondheidsZorg (SBG, Foundation for Benchmarking Mental Healthcare), was established as a trusted third party to inform patients, providers, and insurers on the quality of health care (www.akwaggz.nl). Treatment outcome was deemed the key performance indicator, as “systematic outcomes measurement is the sine qua non of value improvement” (Porter, Larsson, & Lee, 2016).

The nationwide implementation of routine assessment of treatment outcomes in MHS started in 2010, with ROM serving multiple purposes: as clinical support tool, as data source for performance appraisal of providers for patients and financiers, and as data source for scientific research. Assessments at regular intervals (e.g., every 3 months or even session‐by‐session) are required to support treatment, but aggregated pretest and posttest ROM data of treatments are adequate for performance appraisal purposes (www.akwaggz.nl). Patients routinely complete self‐report questionnaires on symptomatology in curative outpatient care, and professionals complete rating scales on their patients' functioning in care for severe mental illness. Providers send their outcome data monthly to the benchmark institute, where data are checked, aggregated, and transformed into various performance indicators (de Beurs et al., 2016; de Beurs et al., 2017). Performance of providers is evaluated by comparing the average pretest‐to‐posttest change in symptoms, functioning, and health‐related quality of life for various patient groups (common mental disorders, severe mental illness, geriatric psychiatry, and substance use disorders) achieved after a year of treatment or after a completed treatment trajectory.

At the start in 2010, a 50% response rate was deemed achievable based on estimates of 70% pretest and 70% posttest inclusion rates (resulting in an overall 49% response rate). Also, we expected that at least 50% response was required for valid aggregated outcome information. This estimate was based on the literature (e.g., Livingston & Wislar, 2012) and on experiences with similar international endeavours, such as the Minnesota Health Scores initiative (www.mnhealthscores.org) and the pay‐for‐performance scheme of the English National Health Service, where a response rate >50% is one of the requirements for providers to qualify for a bonus payment (Gutacker, Street, Gomes, & Bojke, 2015). Dutch MHS providers were allowed 5 years to achieve this 50% response rate, with yearly increments of 10%, and their response rates were monitored by SBG. ROM response rates rose according to the plan. By 2016, 95% of all providers in the Netherlands submitted data of concluded treatments to SBG monthly, and pretest and posttest data of symptomatology and/or functioning were available for 47% of all treatments. There was substantial variance among providers, with some achieving only 20% ROM response and others reaching response rates of 90% or more (www.akwaggz.nl).

We aimed to investigate which factors may bias performance indicators and which response rates yield sufficiently trustworthy information on provider performance. Any response rate below 100% creates the possibility of biased results, as patients with complete data may differ systematically from nonresponders and may not be representative of the entire population of the provider. A comprehensive body of literature has been published on missing data (Graham, 2009; Little & Rubin, 2014; Rubin, 2004; Seaman & White, 2011). Data can be Missing Completely at Random (no relationship between whether a data point is missing and any values within the data set, or any unobserved values). More likely, data are missing systematically, and here, two cases are distinguished: Missing at Random (there is a relationship between missing data points and other observed values in the data set) and Missing Not at Random (there is a relationship between missingness and unobserved data). The latter is nonignorable, as it will bias results (Graham, 2009). An example is endogenous selection bias (i.e., conditioning on a collider; Elwert & Winship, 2014). For instance, some patients may be harder to assess and more difficult to treat too. Consequently, the measured outcomes from more easily assessed patients would overestimate the benefits of MHS treatment, would not be an accurate reflection of the true nationwide results, or would not reflect the true performance of a single MHS provider. Minimization of selection bias or at least information on the extent of bias per provider is essential for the validity and utility of the performance indicator. How much the results are biased depends on the extent of systematic differences between patients with complete and incomplete data. The extent of bias may also depend on the response rate of a provider (Gomes, Gutacker, Bojke, & Street, 2015; Hoenders et al., 2014; Young, Grusky, Jordan, & Belin, 2000). The present study sets out to investigate these issues.

With longitudinal treatment outcome data, selection (accidently or intentionally) can occur at two time points: pretest and posttest. Omission of pretest data may lead to selective *inclusion* bias; omission of posttest data from included subjects may cause selective *attrition* bias. Through selective inclusion at pretest, performance indicators of providers will become positively biased when predominantly patients are assessed for whom a good outcome is expected (e.g., well‐treatable patients with less complex problems, little co‐morbid psychopathology or co‐morbid somatic problems, a first episode, employed, extensive social network, and high socio‐economic status [SES]). Conversely, performance indicators will become negatively biased when mostly difficult‐to‐treat patients are included. An investigation of sample selection bias in ROM data should therefore focus on patient variables with prognostic value for outcome. Selective attrition at posttest will bias outcome towards the positive when patients with unsuccessful therapies are not reassessed. Such bias can be intentionally introduced in the data collection phase but can also occur unintentionally, for instance, because unsuccessfully treated patients (e.g., early dropouts) are less compliant with a posttest assessment.

Intentional or not, we cannot assume that inclusion or attrition occur at random: patients with complete data may differ from the noncomplete group, and countrywide results and findings on the performance of individual providers may become biased by selection. Both inclusion and attrition bias threaten the external validity of the results (Cuddeback, Wilson, Orme, & Combs‐Orme, 2004). Furthermore, the lower the response rate obtained by an MHS provider, the more room there is for biased results.

We investigated the association between patient characteristics (demographic and clinical) and outcome, their association with inclusion and attrition, and their potential to bias aggregated outcomes of providers. Hence, for each provider, we established (a) bias due to selective inclusion at pretest, (b) bias due to selective attrition at posttest, and (c) the combined biasing effect of inclusion and attrition. We also investigated the association between naturally occurring ROM response rates and extent of bias. Based on the findings, we will discuss minimal requirements for inclusion, attrition, and overall response rates to attain sufficiently unbiased performance indicators.

## 2 METHODS

### 2.1 Patient, treatments, and providers

The present study is limited to treatments concluded in 2016 of adult outpatients (aged 18–65) predominantly with common mental disorders, such as mood and anxiety disorders of mild to moderate severity. Other groups of patients were included in this nationwide effort (severe mental disorders, elderly patients, and substance abuse) but different outcomes (and instruments) were used, so these groups are omitted from the present analysis. Mean age of the present sample was X̅ = 38.30 (*SD* = 13.50); 61.1% was female; and 31.9% was treated for depression, 24.5% for an anxiety disorder, and 16.8% for a personality disorder (see Table 1).

**Table 1:** Descriptive statistics of demographic and clinical patient characteristics

Characteristic	M (SD)
Age	38.30 (13.50)
T‐score	51.27 (10.29)
GAF score	53.48 (9.06)
	%
Gender (female)	61.1
Living situation	
Alone	27.8
With partner and children	18.1
With partner, no children	8.3
Alone with children	27.9
Other	17.9
Educational level	
Elementary	7.2
Secondary	25.8
Higher	38.2
Bachelor	20.2
Master or higher	8.6
Main diagnosis (%)	
Mood	31.9
Anxiety	24.5
Personality disorder	16.8
Developmental	11.4
Somatoform	5.9
Eating disorder	2.7
Other	5.9
Urbanization	
Urban (5 levels)	26.5
2	24.7
3	22.3
4	16.3
Rural	10.3
SES (5 levels)	
Low	26.2
2	21.0
3	17.6
4	17.3
High	17.8

Abbreviations: GAF, Global Assessment of Functioning; SES, socio‐economic status.

Treatments were pharmacological, psychosocial (e.g., cognitive–behavioural therapy), or a combination of both, predominantly provided in individual weekly or biweekly sessions with a psychiatrist, clinical psychologist, or psychiatric nurse, as well as in group format. The present study is limited to the first year of treatment; the average duration of treatments was *M* = 42.2; *SD* = 13.4; range = 1–52 weeks.

Providers can be large nationwide‐operating institutions, large institutions providing integrated MHS in a specific region of the country, smaller institutions working locally, or even private practitioners. SBG has contracts with 500+ institutional providers. For the present study, only data were used from institutional providers who submitted at least 25 treatments with complete pre–post data per provider to arrive at a reliable estimate of their performance. This resulted in *n* = 113,707 treatments and *n* = 135 providers. Data of small institutional providers were thus excluded. Individual providers working in private practice were not included in the current data set either.

### 2.2 Demographic and clinical patient variables

Demographic variables included age, gender, living situation, educational level, urbanization level, and SES, the last three variables each in five levels. Clinical variables included pretest T‐score, Global Assessment of Functioning (GAF) score, and primary diagnosis coded according to the Dutch reimbursement system for Diagnosis‐Treatment Combinations, which follows the taxonomy of the Diagnostic and Statistical Manual of Mental disorders ‐ 4th edition (DSM‐IV) (American Psychiatric Association, 2000).

### 2.3 Treatment outcome

To assess severity of symptomatology with common mental disorders, generic self‐report measures were used such as the Brief Symptom Inventory (Derogatis, 1975), the Outcome Questionnaire‐45 (Lambert, Gregersen, & Burlingame, 2004), and the Symptoms Questionnaire‐48 (Carlier et al., 2012). Scores on these self‐report questionnaires were transformed into a common metric (normalized T‐score) with a pretest mean of *T* = 50 (*SD* = 10). Treatment outcome was operationalized as the pretest‐to‐posttest difference in severity of symptoms expressed in T‐scores (ΔT), achieved within the first year of treatment. The average outcome was ΔT = 7.29; *SD* = 10.17.

### 2.4 Threshold values for selective inclusion bias and attrition bias

For selective inclusion or attrition bias, a critical cut‐off value was set at 0.50 ΔT points. As ΔT is based on T‐scores with *SD*_pretest_ = 10, a 0.50 ΔT‐point implies a 0.05 shift in standardized pre–post difference or an effect size of ES = 0.05 (Cohen, 1988; Seidel, Miller, & Chow, 2013). A critical cut‐off value of 0.50 implies that we deem a difference between two providers larger than 1.0 points as not attributable to inclusion or attrition bias.

### 2.5 Statistical analysis

#### 2.5.1 Patient predictors of outcome

We had previously established the prognostic value for posttest level of symptoms in a more extensive set of clinical and demographic variables (Iezzoni, 2013; Warmerdam, de Beurs, Barendregt, & Twisk, 2018), using nonmissing data of the entire sample (*n* = 59,136). Administrative data, irrespective of whether a pretest or a posttest ROM assessment had taken place, were available for almost all patients (95.1%). Only statistically significant demographic and clinical predictors with substantive prognostic power were selected and re‐evaluated as predictors, as variables without influence on outcome are unlikely to bias the performance indicator. The selected variables were analysed with multilevel multivariate regression analysis, incorporating the provider as a level.

Inclusion bias was assessed by comparing demographic and clinical characteristics of patients with and without a pretest score on the outcome measure. We calculated weighted outcome (had all patients been included) by inverse probability weighting (Austin & Stuart, 2015; Rubin, 1997; Seaman & White, 2011). Probability of inclusion was based on multilevel logistic regression analysis, also including the provider as a level in the model. Subsequently, each case got a weight inversely proportional to their chance of inclusion. For example, if personality disorders are underrepresented in the inclusion sample (the subsample with pretest data), all patients with a personality disorder get a weight >1.00 in the analysis. Cases with missing values on the predictors (4.9% of the entire sample) were given a weight of 1.00. Inclusion *bias* was established by comparing actual pretest‐to‐posttest change (ΔT), with estimated change after weighting of cases based on the inverse of the propensity score for inclusion. The estimated change score is reported as ΔT_weighted_.

To estimate attrition bias, missing posttest data were imputed with an estimation of their value based on the pretest score, other prognostic patient characteristics, and provider performance (multilevel imputation; Díaz‐Ordaz, Kenward, Cohen, Coleman, & Eldridge, 2014). To preserve variance in the outcome scores, imputation was repeated five times and the pooled results are reported as ΔT_imputed_ (multiple imputation). Lastly, we combined both sources of bias by estimating outcome after propensity‐of‐inclusion weighting was applied to the data set where missing posttest scores had been imputed (ΔT_combined_).

The extent of bias in the nationwide results and per provider was established by subtracting the ΔTs after propensity weighting of included cases (ΔT_weighted_), imputation of missing posttest data (ΔT_imputed_), and the combined effect of propensity weighting and imputation (ΔT_combined_) from the original ΔT. Positive bias implies overstated results. The association between response rates and extent of inclusion bias, attrition bias, and combined bias is graphically presented in scatterplots and assessed with Pearson correlation coefficients.

## 3 RESULTS

**Figure 1:**
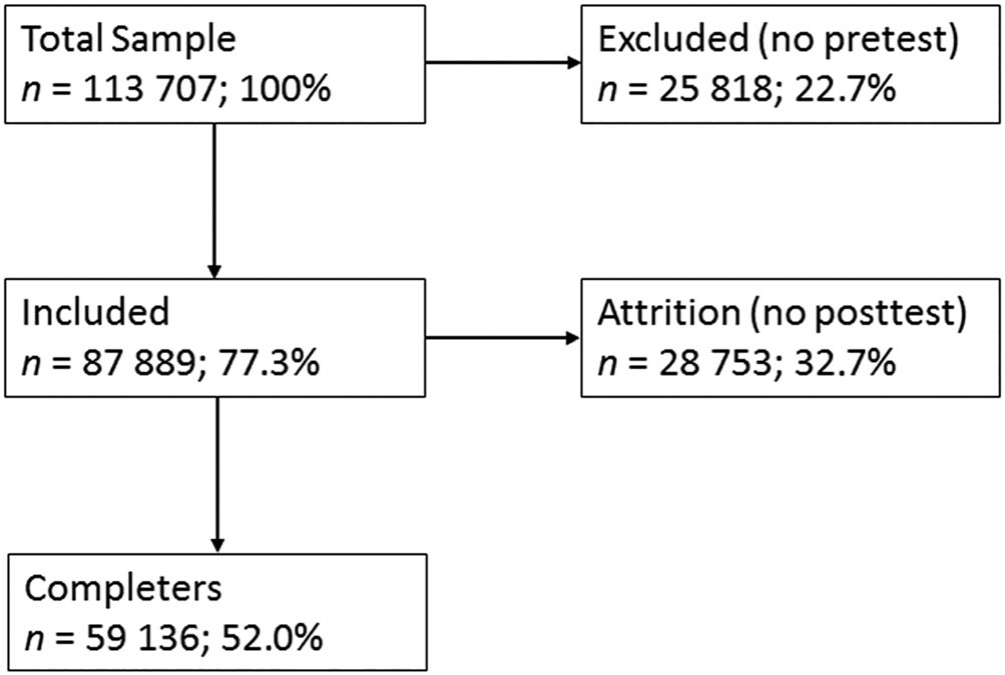
Flowchart for inclusion and attrition of individual patients

Figure 1 presents the loss of data in the present data set due to nonresponse in a flowchart. Data were available from *N* = 113,707 treatments, showing that 25,818 cases were compared with 87,889 cases with pretest data to assess selective inclusion and 59,136 cases were compared with 28,753 cases to assess selective attrition.

To select relevant patient characteristics, we first analysed the bivariate association between demographic and clinical patient variables (predictors) and the posttest score. Most variables had a statistically significant association with outcome, but only five contributed substantially to the multiple model (partial *η* ^2^ > .001, see Table 2). More severe symptomatology (a higher pretest T‐score) and worse functioning (lower GAF score) have the most influence on the posttest score.

**Table 2:** Association of demographic and clinical pretest data with posttest symptomatology based on multilevel analysis

Predictor	β	SE	95% CI	η ^2^
Pretest T‐score	.601*	0.004	[0.593, 0.608]	.311
GAF	−.143*	0.005	[−0.153, −0.130]	.062
SES	−.297*	0.028	[−0.353, −0.241]	.003
Age	.029*	0.003	[0.023, 0.035]	.001
Urbanization	−.161	0.034	[−0.228, −0.095]	.000
Gender	−.426	0.080	[−0.584, −0.270]	.000
Diagnoses				
Personality	1.600*	0.551	[0.520, 2.681]	.011
Mood	−.562	0.547	[−1.634, −0.511]	.000
Anxiety	−.161	0.548	[−1.237, −0.915]	.000
Developmental	−.183	0.554	[−1.270, −0.904]	.000
Somatoform	−.741	0.566	[−1.459, −0.759]	.000
Eating	−.116	0.587	[−1.005, 1.296]	.000
Other	−.908	0.574	[−2.033, 0.216]	.000

Abbreviations: GAF, Global Assessment of Functioning; SES, socio‐economic status.

* β significant at *p* < .0001.

The five predictors with substantial prognostic value for outcome of Table 2 were used to assess selective inclusion and selective attrition in two multiple multilevel logistic regression analyses with binary dependent variables (“included at pretest or not” and “assessed at posttest or not”). Table 3 presents the results. The combination of three prognostic variables predicted inclusion significantly: *χ* ^2^(5) = 1,053.88; *p* < .001. Better functioning (higher GAF score) at pretest, higher SES, and lower age were associated with being included at pretest. These three variables were used to calculate the propensity score.

**Table 3:** Association of demographic and clinical pretest data with inclusion and attrition (multilevel logistic regression analyses)

Predictor	Pretest inclusion				
	β	SE	p	OR	95% CI
Personality	.017	0.020	.380	1.017	[0.850, 1.010]
SES	.049	0.010	.000	1.050	[1.045, 1.055]
GAF	.018	0.001	.000	1.018	[1.017, 1.020]
Age	−.001	0.001	.044	0.999	[0.998, 1.000]

	Posttest attrition				
	β	SE	p	OR	95% CI
Personality	.073	0.020	.000	1.076	[1.036, 1.116]
SES	.042	0.005	.000	1.043	[1.010, 1.090]
GAF	.011	0.001	.000	1.011	[1.009, 1.013]
Age	.007	0.001	.000	1.007	[1.006, 1.009]
Pretest T‐score	−.001	0.001	.372	0.999	[0.998, 1.000]

Abbreviations: GAF, Global Assessment of Functioning; SES, socio‐economic status.

Having a personality disorder, higher SES, better functioning (higher GAF score), and higher age were associated with attrition at posttest; the pretest T‐score (the most prominent predictor of the posttest score) was not associated with attrition. The combination of the four variables predicted attrition significantly: *χ* ^2^(6) = 656.45; *p* < .001. These four variables and the pretest score (all variables that appeared predictive of outcome in Table 2) were used to estimate missing posttest scores for imputation. The multilevel estimation of propensity and imputation included one additional level: the provider.

### 3.1 Analysis of patient‐based data

Next, mean ΔT before and after imputation and before and after inverse propensity weighting was established. On the *Per patient* columns, Table 4 presents the average ΔT score for *n* = 59,136 cases with complete data (ΔT), after weighting cases based on the inverse propensity score for inclusion (ΔT_weighted_), after imputation of missing posttest scores (ΔT_imputed_), and after both weighting and imputation of missing posttest scores (ΔT_combined_). Overall, weighting has a greater effect on the average ΔT than imputing missing posttest scores. Weighting of cases affects the mean ΔT substantially: ΔT_weighted_ is 0.95 points higher than ΔT was, indicating that the original ΔT was a slight underestimation. Attrition bias was smaller and in the other direction: ΔT_imputed_ is only 0.11 points lower than the originally achieved ΔT. The combined effect of inclusion and attrition bias is 0.77 points. This suggest that the results based on incomplete data slightly underestimate the true treatment outcome for the average patient.

**Table 4:** Results of patient‐based and provider‐based analyses of data, demonstrating the extent of bias in average ΔTs

Performance indicator	Per patient			Per provider (N = 135)			
	N	X̅	SD	X̅	SD	Minimum	Maximum
ΔT	59,136	7.29	10.17	7.19	2.73	−0.62	15.34
ΔT_weighted_	113,707	8.24	10.39	7.18	2.72	−0.55	15.19
ΔT_imputed_	87,889	7.18	10.20	7.16	2.59	−0.05	14.69
ΔT_combined_	113,707	8.06	10.39	7.17	2.57	−0.10	14.64

Abbreviations: ΔT, difference between pretest and posttest T‐score; ΔT_combined_, difference between pretest and posttest after imputation of missing posttest scores and inverse propensity weighting; ΔT_imputed_, difference between pretest and posttest after imputation of missing posttest scores; ΔT_weighted_, difference between pretest and posttest adjusted by inverse propensity weighting.

### 3.2 Analysis of provider‐based data

At least 25 treatments with complete data were submitted by 135 providers. The largest provider contributed 10,303 cases, the smallest 25. The median number of treatments was *M* = 141. We assessed selective inclusion and attrition bias per provider and calculated the mean ΔT of all providers, based on their average performance.

Selection was substantial: the mean percentage of included patients at pretest was 77% (range among providers: 19–99%). However, selective inclusion did not bias the average outcome substantially (see Table 4, *Per provider* columns).

There was considerable attrition too. The mean percentage of included patients who were reassessed after 1 year was 66% (range among providers: 25–92%), but again, this did not bias the overall mean performance of all providers. The overall response rate after selection due to inclusion and attrition was 52% (range 11–90%). All differences between the various mean ΔTs on the *Per patient* columns of Table 4 are small and not statistically significant (*T* < 0.95; *p* > .34), and the countrywide provider‐based average is not affected by selection bias.

### 3.3 Bias per provider

**Figure 2:**
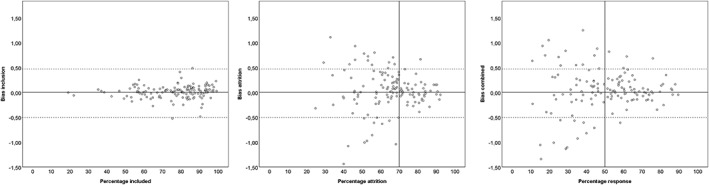
Scatterplots of bias due to selective inclusion, selective attrition, and bias due to all combined by response percentage. Points represent the response rate of providers (x‐axis) by the extent of the bias (y‐axis) in ΔT units; positive bias implies overstated results, negative bias understated results

Bias due to selective inclusion ranged from −0.53 to 0.49 among providers: only one provider had a bias of ±0.50 or more, and 10 providers (7.4%) had a bias of ±0.25 or more (see Table 5 and Figure 2). There was no relation between the percentage of included patients and absolute inclusion bias (*r* = −.10, *n* = 135, *p* = .27). Bias due to attrition ranged from −1.44 to 1.25 among providers: 30 providers (22.2%) had a bias of ±0.50 or more, and 15 providers (11.1%) had a bias of ±0.70 or more. The association between the percentage of missing posttests and absolute attrition bias was substantial: *r* = .48, *n* = 135, *p* < .001. Bias due to the combined effect of inclusion and attrition ranged from −1.53 to 1.25 among providers: 29 providers (21.5%) had a bias of ±0.50, and 13 providers (9.6%) had a bias ±0.70. There was again a strong association between overall response and absolute bias: *r* = .50, *n* = 135, *p* < .001. Bias went both ways: 16 providers had positively biased results (ΔT > ΔT_combined_), and 13 providers had negatively biased results (ΔT > ΔT_combined_).

**Table 5:** Number (%) of providers with inclusion, attrition, and combined bias beyond set limits

	Bias^a^		Absolute bias
Criterion	≤−.50	≥.50	≥.50
Inclusion	1 (0.7)	0 (0.0)	1 (0.7)
Attrition	13 (9.6)	17 (12.6)	30 (22.2)
Combined	13 (9.6)	16 (11.9)	29 (21.5)

a Bias calculated as ΔT minus ΔT_weighted_, ΔT _imputed_, or ΔT _combined_; positive bias implies overstated results.

## 4 DISCUSSION

In this paper, we presented a strategy to estimate both inclusion and attrition bias in ROM data to assess provider performance. Included and nonincluded patients at pretest did not differ on most demographic and clinical variables. Although some statistically significant differences were found, the size of their effect (bivariate and multiple) on outcome was small and close to zero. Nevertheless, in the patient‐based analyses, selection bias was found, predominantly due to selective inclusion. Also, selective attrition at posttest biased the results, here most visibly in the results of provider‐based analyses. These findings stress the value of distinguishing both sources of bias: selective inclusion and selective attrition. Finally, the results reveal that an overall response rate of 50% adequately safeguards against biased provider‐based results.

When analysing patient‐based data, the nationwide average was more biased by selective inclusion than by attrition. The elevated ΔT_weighted_ is most likely due to giving more weight to cases with a lower level of functioning (GAF scores are negatively associated with pre–post gain). However, the biasing effect of selective inclusion was less pronounced when inspecting the results per provider. Provider‐based data also demonstrated effect of selection, but bias went in both directions, with half of the providers increasing their ΔT and the other half decreasing it. Apparently, the two effects even each other out in the provider‐based nationwide average. These findings taken together suggest that selective inclusion is potentially a strong biasing factor, but institutes do not appear to selectively include patients in ROM. For only one provider, statistical correction for selective inclusion led to a substantially lower ΔT (bias > −.50); for all other providers, inclusion bias remained within critical limits. Finally, there is no association between inclusion rate and bias; a low inclusion rate does not appear to lead to biased results. These findings seem contradictory but may be explained by providers' current motivation to optimize inclusion rates through incentives from their financiers. No financing of MHS based on outcomes has been implemented yet in the Netherlands. Consequently, providers are motivated to assess as many patients as possible rather than exclude potentially hard‐to‐treat patients from pretest assessment and thus keep unsuccessful treatments out of view. Hence, although selective inclusion can potentially bias outcome results strongly, it appears to be a factor with only limited effect in current performance monitoring among providers in the Netherlands.

The patient‐based analysis on selective attrition at posttest showed that imputation of missing data had only a limited effect on the countrywide average, but a provider‐based analysis revealed biased results for *n* = 30 providers (22.2%) due to selective attrition. Moreover, a strong association is found between the extent of posttest attrition and absolute bias. As previously mentioned, providers are incentivized for response rates and not for outcomes. Consequently, attrition is most likely due to unwillingness of patients to comply with reassessments when their treatment was unsuccessful rather than a result of intentionally not assessing unsuccessful treatments at posttest. For instance, a substantial proportion of patients terminate their treatment at an early stage, usually after one or two sessions (Swift & Greenberg, 2012), and it is difficult to obtain posttest data from these patients.

Statistical correction for both selective sampling mechanisms, by weighting cases based on their propensity for inclusion and by imputing posttest scores, exposed 29 providers with biased results. Interestingly, bias went both ways, with some providers having better outcomes after statistical correction (*n* = 16, 11.9%) and some having worse outcomes (*n* = 13, 9.6%). Some providers may selectively include patients who are more likely to have a positive outcome or selectively leave failed treatments unassessed, leading to a positively biased outcome indicator. Interestingly, a similar proportion of providers had negatively biased results, possibly due to overinclusion of hard‐to‐treat patients or a selective overrepresentation at posttest of failed treatments. The present data were collected in a context where providers were incentivized to obtain high response rates. Results may be different if providers are incentivized towards better performance, for example, by a pay‐for‐performance scheme. This may encourage exclusion of hard‐to‐treat cases at pretest and exclusion of failed treatments at posttest, resulting in biased performance indicators and exaggerating what has been achieved in treatment. With the present data‐analytic approach, we can reveal the extent of this bias and flag overstated performance.

Close inspection of Figure 2 reveals that the extent of inclusion bias was limited; for most providers, bias due to selective inclusion fell between critical limits of ±0.50. Attrition had a more profound biasing effect, and a minimum 70% response is required to keep bias within critical limits. When both effects are combined, the graph suggests that at least 50% response is required to keep bias sufficiently at bay. When a 50% response rate was achieved, only four providers (3.0%) remained biased due to the combined effect of selective inclusion and attrition beyond the stringent limit of 0.5 ΔT points. However, it should be noted that currently, only 62 (45.9%) of the providers achieve this level of completeness of data.

The present findings suggest that 100% implementation of ROM is not needed to obtain valid information on providers' performance, as weighting for noninclusion and imputation of missing posttest scores yields similar results for most providers on the performance indicator. A posttest inclusion rate of 70% and an overall response rate of at least 50% seems sufficient for a representative estimate of a provider's performance. This 50% response rate coincides with required levels of the pay‐for‐performance scheme of the British National Health Service (Gutacker et al., 2015). For providers with a response rate under 50%, results on their performance became untrustworthy, as chances are that their results were more than 0.5 ΔT points off the mark. The lower the overall response rate, the larger the bias and the more bias went both ways: some providers scored higher and some lower after correcting for selection bias.

The findings support the initial decision from 2010 to recommend 50% response as a minimum requirement for providers, in order to deem their results sufficiently unbiased by selection. There are other reasons to strive for optimum implementation of ROM beyond the 50% mark and to encourage providers to improve data collection. First of all, ROM was not primarily implemented for accountability but predominantly intended as a beneficial adjunct to treatment (de Beurs, Barendregt, & Warmerdam, 2017). Research has shown that ROM by itself can lead to better results (Boswell, Kraus, Miller, & Lambert, 2013), especially for patients at risk for treatment failure (Lambert, 2010). Treatment failures tend to be overlooked by therapists (Hannan et al., 2005). ROM can also lead to more efficient treatment delivery as outcome feedback may reduce treatment length (Delgadillo et al., 2017). Furthermore, ROM grants patients a more active role in their own recovery (Patel, Bakken, & Ruland, 2008). All patients deserve this adjunct to treatment, not merely a fraction to ensure a nonbiased performance indicator. Hence, striving for high ROM response rates is good clinical practice, but it is also vital to counteract providers who game the system (Bevan & Hood, 2006) by biasing results through deliberate pretest or posttest selection of treatments with actual or expected positive outcomes.

The general aim of ROM and benchmarking (continuous improvement of MHS quality) is underwritten by all stakeholders, but benchmarking is still a controversial element, especially for those who fear negative financial consequences. Opponents criticize the validity or value of performance indicators based on ROM (van Os et al., 2012) and echo warnings for perverse consequences, such as cherry‐picking the best treatable patients (Killaspy, 2018) or gaming the system by other means (Jacobs, 2014). Others warn of adverse effects on the clinical application of ROM if data are also used for benchmarking (Delespaul, 2015). Indeed, some Dutch providers have implemented a minimal variant of ROM, measuring only at pretest and posttest, which enables submitting data to SBG but does not support clinical decision making during treatment. The controversy regarding the various ambitions with ROM resulted in a heated debate in the Netherlands, and selection bias is only one threat to the validity of benchmarking data and other issues are still unresolved. Thus, the discussion is ongoing about the usefulness of gathering nationwide outcome data, focusing on patients' privacy and the suitability of outcome data to serve as a quality indicator of treatment. Currently, a new foundation (Stichting Akwa GGZ) is charged with finding a better balance between these two aims and will revise the system to increase its usefulness to plan, monitor, and modify individual treatment. The selection of allowed outcome measures will be broadened to include disorder‐specific measures, and guidelines for the frequency of ROM assessments will be offered. Patients will be asked to explicitly consent to the use of their ROM data for quality monitoring. Publication of providers' aggregated outcomes will become voluntary and depend on their contract with insurers.

### 4.1 Strengths

There are various approaches to imputation of missing posttest data to choose from (Díaz‐Ordaz et al., 2014), depending on whether differential provider effects are taken into account (Taljaard, Donner, & Klar, 2008) or not: multiple (single‐level) regression analysis and multilevel regression analysis. Multiple regression estimation of missing posttest scores is based on pretest information on demographic and clinical characteristics of patients. Basically, missing posttest data are estimated on what is achieved on average with other patients with similar characteristics. However, this estimate does not take into account the performance of the provider. By contrast, imputation based on *multilevel* analysis does consider provider performance (based on complete pretest‐to‐posttest data). Consequently, the single‐level regression‐based approach diminishes differences between providers, whereas the multilevel approach takes these differences into account. As there is considerable variation in outcomes among providers, the multilevel approach was deemed the most appropriate, even though the more stringent approach yielded a verdict of biased results more readily. It is nonetheless wise to set the bar high for validity of performance indicators and better to err on the side of deeming them biased rather than too readily deeming them valid.

It is not uncommon to assess selection bias by comparing subjects with complete data to all other subjects with incomplete data, irrespective of when data loss occurred (Gomes et al., 2015). However, in a longitudinal study design, data loss can occur at two distinct time points: the pretest and the posttest. In the present study, selection bias was divided into two potential sources of bias: selective inclusion and selective attrition, and effects of both were investigated separately. Different variables were associated with loss of data at pretest and at posttest. According to patient‐based analyses, selective inclusion had a greater biasing effect than selective attrition. This is a fortunate finding because it is easier for a provider to counteract data loss at pretest than at posttest. At pretest, most patients are willing to meet demands for information on the severity of their illness or other variables. At posttest, it may be more difficult to obtain this information, especially from patients who discontinue treatment prematurely. This may limit the window of opportunity to get such data. Consequently, bias due to attrition is harder to prevent than bias due to selection.

This paper offers a strategy for providers who want to know the trustworthiness of their aggregated ROM data and want to establish whether their outcomes are biased by selective inclusion or attrition. Multiple imputation and weighting will give a methodologically sound estimate of the extent and direction of potential bias, even though multilevel analysis is not feasible with data from a single provider. However, the underlying analyses may be too complicated or cumbersome for many providers. For them, the present results do offer a simple message: ensure that you have a high response rate (>50%) and the risk of biased results will be limited. When the response rate falls below 50%, ΔT might be biased and misleading as performance indicator.

### 4.2 Limitations

A limitation of this study is that we restricted our sample to adult patients with common mental disorders. Findings may be different for other age groups or disorder groups, and this still needs to be investigated. For instance, with severe mental disorders, we assess treatment outcome with the HoNOS (Wing et al., 1998), a rating scale usually completed by a professional. Then, the professional, not the patient, is the source of the data, and consequently nonresponse is due to lack of compliance of professionals with administrative processes, like completion of the HoNOS. Under such conditions, nonrandom missingness may be even more likely. Furthermore, in the Netherlands, treatment outcome is evaluated at least once a year, and for the present study, we used outcome data from the first year of treatment. This means that longer treatment trajectories were only partially evaluated.

Weighting and imputation of posttest data was based on a selective set of demographic and clinical variables. Various other factors such as work status or ethnic background may be relevant, but these data were not available to us. Further research should include additional factors that are potentially associated with outcome and missingness of outcome data. In addition, interactions between observed demographic and clinical variables may be relevant to outcome. For instance, the effect of age or gender may differ between diagnostic groups. Interactions among predictors were not included in the present study, as they would complicate a subject that is already difficult to understand.

To assess selective inclusion, an alternative approach to inverse probability weighting would have been multiple imputation of missing data. Based on patient characteristics, a *pretest* score could have been estimated and imputed as well. Based on this imputed pretest score and other patient characteristics, a posttest score can be estimated and imputed. We decided against this double imputation, as it would have multiplied the uncertainty about the resulting score and ΔT. We do not know of studies where this double imputation process has been applied and evaluated.

## 5 CONCLUSION AND IMPLICATION

If response rates fall below 50%, there is a substantial chance of aggregated outcomes of providers being biased by selective inclusion or attrition. We propose using two extra indicators per provider for the trustworthiness of their performance indicator: the overall response rate (>50% or not) and whether the results are unbiased by the combination of selective inclusion and attrition (<0.5 ΔT difference or <0.05 ES between weighted posttest imputed performance and observed performance). Accordingly, the representativeness of the outcome data will be adequately revealed.

## CONFLICT OF INTERESTS

The authors have no conflict of interest.

## References

[cpp2364-bib-0001] American Psychiatric Association (2000). Diagnostic and statistical manual of mental disorders, text revision; DSM‐IV‐TR. Washington, DC: Author.

[cpp2364-bib-0002] Austin, P. C. , & Stuart, E. A. (2015). Moving towards best practice when using inverse probability of treatment weighting (IPTW) using the propensity score to estimate causal treatment effects in observational studies. Statistics in Medicine, 34, 3661–3679. 10.1002/sim.6607 26238958 PMC4626409

[cpp2364-bib-0045] Bayney, R. (2005). Benchmarking in mental health: An introduction for psychiatrists. Advances in Psychiatric Treatment, 11, 305–314. 10.1192/apt.11.4.305

[cpp2364-bib-0003] Bevan, G. , & Hood, C. (2006). What is measured is what matters: Targets and gaming in the English public health care system. Public Administration, 84, 517–538. 10.1111/j.1467-9299.2006.00600.x

[cpp2364-bib-0004] Boswell, J. F. , Kraus, D. R. , Miller, S. D. , & Lambert, M. J. (2013). Implementing routine outcome monitoring in clinical practice: Benefits, challenges, and solutions. Psychotherapy Research, 25, 6–19. 10.1080/10503307.2013.817696 23885809

[cpp2364-bib-0005] Carlier, I. , Schulte‐Van Maaren, Y. , Wardenaar, K. , Giltay, E. , Van Noorden, M. , Vergeer, P. , & Zitman, F. (2012). Development and validation of the 48‐item Symptom Questionnaire (SQ‐48) in patients with depressive, anxiety and somatoform disorders. Psychiatry Research, 200, 904–910. 10.1016/j.psychres.2012.07.035 22884307

[cpp2364-bib-0006] Cohen, J. (1988). Statistical power analysis for the behavioural sciences. Hillsdale, NJ: Lawrence Erlbaum Associates.

[cpp2364-bib-0007] Cuddeback, G. , Wilson, E. , Orme, J. G. , & Combs‐Orme, T. (2004). Detecting and statistically correcting sample selection bias. Journal of Social Service Research, 30(3), 19–33. 10.1300/J079v30n03_02

[cpp2364-bib-0008] de Beurs, E. , Barendregt, M. , de Heer, A. , van Duijn, E. , Goeree, B. , Kloos, M. , … Merks, A. (2016). Comparing methods to denote treatment outcome in clinical research and benchmarking mental health care. Clinical Psychology & Psychotherapy, 23, 308–318. 10.1002/cpp.1954 25847057

[cpp2364-bib-0009] de Beurs E., Barendregt M., & Warmerdam L. (Eds.) (2017). Behandeluitkomsten: bron voor kwaliteitsbeleid in de GGZ [Treatment outcome: Source of quality management in mental Health Care]. Amsterdam: Boom.

[cpp2364-bib-0010] de Beurs, E. , den Hollander‐Gijsman, M. E. , van Rood, Y. R. , van der Wee, N. J. , Giltay, E. J. , van Noorden, M. S. , … Zitman, F. G. (2011). Routine outcome monitoring in the Netherlands: Practical experiences with a web‐based strategy for the assessment of treatment outcome in clinical practice. Clinical Psychology & Psychotherapy, 18, 1–12. 10.1002/cpp.696 20238371

[cpp2364-bib-0011] de Beurs, E. , Warmerdam, E. H. , Oudejans, S. C. C. , Spits, M. , Dingemanse, P. , de Graaf, S. , … van Son, G. E. (2017). Treatment outcome, duration, and costs: A comparison of performance indicators using data from eight mental health care providers in the Netherlands. Administration and Policy in Mental Health and Mental Health Services Research, 45, 212–223. 10.1007/s10488-017-0818-x PMC5809571 28735344

[cpp2364-bib-0012] Delespaul, P. A. (2015). Routine outcome measurement in the Netherlands—A focus on benchmarking. International Review of Psychiatry, 27, 320–328. 10.3109/09540261.2015.1045408 26107997

[cpp2364-bib-0013] Delgadillo, J. , Overend, K. , Lucock, M. , Groom, M. , Kirby, N. , McMillan, D. , … de Jong, K. (2017). Improving the efficiency of psychological treatment using outcome feedback technology. Behaviour Research and Therapy, 45, 170–184. 10.1016/j.brat.2017.09.011 29024821

[cpp2364-bib-0014] Derogatis, L. R. (1975). The Brief Symptom Inventory. Baltimore, MD: Clinical Psychometric Research.

[cpp2364-bib-0015] Díaz‐Ordaz, K. , Kenward, M. G. , Cohen, A. , Coleman, C. L. , & Eldridge, S. (2014). Are missing data adequately handled in cluster randomised trials? A systematic review and guidelines. Clinical Trials, 11, 590–600. 10.1177/1740774514537136 24902924

[cpp2364-bib-0016] Elwert, F. , & Winship, C. (2014). Endogenous selection bias: The problem of conditioning on a collider variable. Annual Review of Sociology, 40, 31–53. 10.1146/annurev-soc-071913-043455 PMC6089543 30111904

[cpp2364-bib-0017] Enthoven, A. C. , & van de Ven, W. P. M. M. (2007). Going Dutch—Managed‐competition health insurance in the Netherlands. New England Journal of Medicine, 357, 2421–2423. 10.1056/NEJMp078199 18077805

[cpp2364-bib-0018] Gomes, M. , Gutacker, N. , Bojke, C. , & Street, A. (2015). Addressing missing data in patient‐reported outcome measures (PROMS): Implications for the use of PROMS for comparing provider performance. Health Economics, 25, 515–528. 10.1002/hec.3173 25740592 PMC4973682

[cpp2364-bib-0019] Graham, J. W. (2009). Missing data analysis: Making it work in the real world. Annual Review of Psychology, 60, 549–576. 10.1146/annurev.psych.58.110405.085530 18652544

[cpp2364-bib-0020] Gutacker, N. , Street, A. , Gomes, M. , & Bojke, C. (2015). Should English healthcare providers be penalised for failing to collect patient‐reported outcome measures? A retrospective analysis. Journal of the Royal Society of Medicine, 108, 304–316. 10.1177/0141076815576700 25827906 PMC4535435

[cpp2364-bib-0021] Hannan, C. , Lambert, M. J. , Harmon, C. , Nielsen, S. L. , Smart, D. W. , Shimokawa, K. , & Sutton, S. W. (2005). A lab test and algorithms for identifying clients at risk for treatment failure. Journal of Clinical Psychology in Medical Settings, 61, 155–163. 10.1002/jclp.20108 15609357

[cpp2364-bib-0022] Hoenders, R. H. , Bos, E. H. , Bartels‐Velthuis, A. A. , Vollbehr, N. K. , van der Ploeg, K. , de Jonge, P. , & de Jong, J. T. (2014). Pitfalls in the assessment, analysis, and interpretation of routine outcome monitoring (ROM) data: Results from an outpatient clinic for integrative mental health. Administration and Policy in Mental Health and Mental Health Services Research, 41, 647–659. 10.1007/s10488-013-0511-7 23884455

[cpp2364-bib-0023] Iezzoni L. (Ed.) (2013). Risk adjustment for health care outcomes (4th ed.). Chicago, Ill: Health Administration Press.

[cpp2364-bib-0024] Jacobs, R. (2014). Payment by results for mental health services: Economic considerations of case‐mix funding. Advances in Psychiatric Treatment, 20, 155–164. 10.1192/apt.bp.113.011312

[cpp2364-bib-0025] Kilbourne, A. M. , Beck, K. , Spaeth‐Rublee, B. , Ramanuj, P. , O'Brien, R. W. , Tomoyasu, N. , & Pincus, H. A. (2018). Measuring and improving the quality of mental health care: A global perspective. World Psychiatry, 17, 30–38. 10.1002/wps.20482 29352529 PMC5775149

[cpp2364-bib-0026] Killaspy, H. (2018). Improving the quality of global mental health care requires universal agreement on minimum national investment. World Psychiatry, 17, 40–41. 10.1002/wps.20484 29352550 PMC5775141

[cpp2364-bib-0027] Lambert, M. J. (2007). Presidential address: What we have learned from a decade of research aimed at improving psychotherapy outcome in routine care. Psychotherapy Research, 17, 1–14. 10.1080/10503300601032506

[cpp2364-bib-0028] Lambert, M. J. (2010). Prevention of treatment failure. The use measuring, monitoring, and feedback in clinical practice. Washington, D.C.: American Psychological Association.

[cpp2364-bib-0029] Lambert, M. J. , Gregersen, A. T. , & Burlingame, G. M. (2004). The Outcome Questionnaire‐45 In Maruish M. E. (Ed.), The use of psychological testing for treatment planning and outcomes assessment: Volume 3: Instruments for adults (3rd ed) (pp. 191–234). Mahwah, NJ US: Lawrence Erlbaum Associates Publishers.

[cpp2364-bib-0030] Little, R. J. , & Rubin, D. B. (2014). Statistical analysis with missing data (Vol. 333). Hoboken: John Wiley & Sons.

[cpp2364-bib-0031] Livingston, E. H. , & Wislar, J. S. (2012). Minimum response rates for survey research. Archives of Surgery, 147, 110–110. 10.1001/archsurg.2011.2169 22351903

[cpp2364-bib-0032] Patel, S. R. , Bakken, S. , & Ruland, C. (2008). Recent advances in shared decision making for mental health. Current Opinion in Psychiatry, 21, 606–612. 10.1097/YCO.0b013e32830eb6b4 18852569 PMC2676935

[cpp2364-bib-0033] Porter, M. E. , Kaplan, R. S. , & Frigo, M. L. (2017). Managing health care costs and value. Strategic Finance, 98, 24.

[cpp2364-bib-0034] Porter, M. E. , Larsson, S. , & Lee, T. H. (2016). Standardizing patient outcomes measurement. New England Journal of Medicine, 374, 504–506. 10.1056/NEJMp1511701 26863351

[cpp2364-bib-0035] Rubin, D. B. (1997). Estimating causal effects from large data sets using propensity scores. Annals of Internal Medicine, 127, 757–763. 10.7326/0003-4819-127-8_Part_2-199710151-00064 9382394

[cpp2364-bib-0036] Rubin, D. B. (2004). Multiple imputation for nonresponse in surveys (Vol. 81). New York: John Wiley & Sons.

[cpp2364-bib-0037] Seaman, S. R. , & White, I. R. (2011). Review of inverse probability weighting for dealing with missing data. Statistical Methods in Medical Research, 22, 278–295. 10.1177/0962280210395740 21220355

[cpp2364-bib-0038] Seidel, J. A. , Miller, S. D. , & Chow, D. L. (2013). Effect size calculations for the clinician: Methods and comparability. Psychotherapy Research, 24, 470–484. 10.1080/10503307.2013.840812 24188906

[cpp2364-bib-0039] Swift, J. K. , & Greenberg, R. P. (2012). Premature discontinuation in adult psychotherapy: A meta‐analysis. Journal of Consulting and Clinical Psychology, 80, 547–559. 10.1037/a0028226 22506792

[cpp2364-bib-0040] Taljaard, M. , Donner, A. , & Klar, N. (2008). Imputation strategies for missing continuous outcomes in cluster randomized trials. Biometrical Journal, 50, 329–345. 10.1002/bimj.200710423 18537126

[cpp2364-bib-0041] van Os, J. , Kahn, R. , Denys, D. , Schoevers, R. A. , Beekman, A. T. , Hoogendijk, W. J. , … Leentjens, A. F. (2012). ROM: Gedragsnorm of dwangmaatregel? Overwegingen bij het themanummer over routine outcome monitoring [Behavioural standard or coercive measure? Some considerations regarding the special issue on ROM]. Tijdschrift voor Psychiatrie, 54, 245–253. TVPart_9345 22422417

[cpp2364-bib-0042] Warmerdam, L. , de Beurs, E. , Barendregt, M. , & Twisk, J. W. (2018). Comparing single‐level and multilevel regression analysis for risk adjustment in Dutch mental health care. Journal of Public Health, 26, 1–7. 10.1007/s10389-018-0921-9 29416959

[cpp2364-bib-0043] Wing, J. K. , Beevor, A. S. , Curtis, R. H. , Park, S. B. , Hadden, S. , & Burns, A. (1998). Health of the Nation Outcome Scales (HoNOS). Research and development. British Journal of Psychiatry, 172, 11–18. 10.1192/bjp.172.1.11 9534825

[cpp2364-bib-0044] Young, A. S. , Grusky, O. , Jordan, D. , & Belin, T. R. (2000). Routine outcome monitoring in a public mental health system: The impact of patients who leave care. Psychiatric Services, 51, 85–91. 10.1176/ps.51.1.85 10647138

